# Creating a neuro-oncology framework for an empowered and engaged peer volunteer patient community

**DOI:** 10.1093/nop/npaf119

**Published:** 2025-11-18

**Authors:** Naomi Hoffer, Eduardo Rodriguez Almaraz, Lacy Fetting, Jennie Taylor, Susan Chang

**Affiliations:** Division of Neuro-Oncology, Department of Neurological Surgery, University of California, San Francisco (N.H., E.R.A., L.F., J.T., S.C.); Division of Neuro-Oncology, Department of Neurological Surgery, University of California, San Francisco (N.H., E.R.A., L.F., J.T., S.C.); Division of Neuro-Oncology, Department of Neurological Surgery, University of California, San Francisco (N.H., E.R.A., L.F., J.T., S.C.); Division of Neuro-Oncology, Department of Neurological Surgery, University of California, San Francisco (N.H., E.R.A., L.F., J.T., S.C.); Division of Neuro-Oncology, Department of Neurological Surgery, University of California, San Francisco (N.H., E.R.A., L.F., J.T., S.C.)

**Keywords:** brain tumors, community support, peer-to-peer support, supportive care, survivorship

## Abstract

**Background:**

Brain tumor patients often face isolation, identity loss, and existential distress. Peer-to-peer support can address these challenges, yet little guidance exists for supporting neuro-oncology patient volunteers who provide such care. Herein we describe our institutional experience with an innovative program that supports volunteers and provides for peer-to-peer initiatives in neuro-oncology.

**Methods:**

In 2020, the UCSF Sheri Sobrato Brain Cancer Survivorship Program launched a peer support initiative pairing brain tumor patients with trained volunteer peers. To support volunteers, the program offers an optional ongoing community facilitated by a cancer coach trainer, a neuropsychologist, and a social worker. Weekly sessions included training, check-ins, group discussions, feedback opportunities, and an annual feedback survey.

**Results:**

Between August 2020 and October 2023, six cohorts (46 peer volunteers) were trained, with a total of 132 peer matches made for 116 patient requests. Most volunteers (70%) attended 11 or more weekly meetings. All respondents to the annual feedback survey (*n* = 30) expressed high satisfaction with the program. Volunteers highlighted benefits including mutual support, a sense of community, meaningful contributions, personal growth, and empowerment.

**Conclusion:**

In our experience, patient peer volunteer programs show promise in reducing isolation and restoring purpose. This unique 3-year pilot suggests that ongoing group support and training are a plausible and effective way to enhance a peer-to-peer volunteer program. Future efforts will focus on developing a structured curriculum for neuro-oncology patient peer volunteers and prospective evaluation of the impact of the program.

Key Points

- Peer programs in neuro-oncology offer vital emotional support to complement clinical care.
- Structured peer support fosters resilience and purpose, empowering the “Thriver” identity.

Importance of the Study Traditional patient-driven peer support programs often overlook the unique complexities faced by individuals with brain tumors, including cognitive changes, uncertainty, and profound feelings of isolation. We propose a framework that trains survivors to serve as peer volunteers while also supporting them throughout their own ongoing cancer journey. By fostering meaningful connections, emotional resilience, and a sense of purpose, our experience with this model shows that it empowers peer volunteers to engage in a way that benefits both themselves and others. Importantly, the framework acknowledges the emotional demands placed on volunteers, offering structured training, ongoing support, and an identity-affirming community through the Thrivers program. This approach demonstrates how peer-to-peer models can be successfully adapted to high-acuity clinical settings, providing a scalable and sustainable strategy that enhances the well-being of both patients and peer volunteers.

Primary malignant central nervous system (CNS) tumors occur at a global incidence rate of 3.5 per 100,000 persons.^1^ According to the American Cancer Society, it is estimated that 24,820 adults will be diagnosed with primary brain and spinal cord tumors in 2025.^2^ The prognosis is often poor, with a five-year survival rate of 35.6% across all brain tumors and just 6.8% for glioblastoma, the most common malignant brain tumor. Beyond the medical burden, individuals with brain cancer face significant psycho-social challenges, including isolation, existential distress, and role loss.^3^

Living with a brain tumor presents as a complex interplay between psychological, emotional, and physical challenges that extend beyond medical treatment. While research on the psycho-social burden of this population remains limited, existing studies highlight that survivors often experience feelings of loneliness, strained relationships, and diminished purpose in life.^4^^,^^5^ Moreover, the cumulative effects of cognitive decline, changes in mood, and physical symptoms often lead to withdrawal from previously fulfilling roles, such as professional, social, and family responsibilities, further exacerbating feelings of isolation and distress.^6^^,^^7^ The chronic symptom burden and long-term survivorship challenges highlight the necessity of an innovative and sustainable psychosocial support model to address these unmet needs.^8^

Peer-to-peer support is a process in which individuals with shared experiences provide emotional, social, and practical support to one another. Within healthcare, it is widely utilized to help individuals manage chronic illnesses, mental health conditions, substance use disorders, and disabilities.^9^^,^^10^ In oncology and other medical settings, peer support involves individuals facing similar health challenges offering guidance, reassurance, and shared coping strategies.

Peer-to-peer support fulfills several functions that traditional healthcare services may not provide, offering socio-emotional support, strategies for daily life management, and motivation to engage in positive health behaviors.^8^ Peer supporters, by virtue of their lived experience, create a non-hierarchical and reciprocal relationship with those seeking support, making them uniquely positioned to complement formal care.

Although definitions, structure, and evaluation of peer-to-peer support programs vary widely,^10^ evidence suggests that they provide significant benefits, including reduced isolation and loneliness, improved emotional well-being, and enhanced coping skills.^11–13^ Studies in neuro-oncology suggest that even a single meeting with a peer can offer meaningful support and reassurance.^13^ Peer-to-peer support can provide hope and guidance to those receiving support, particularly for newly diagnosed survivors, while also fostering self-efficacy, potentially reducing the burden on family caregivers.^14^

However, the willingness of current patients to support peers with brain tumors might be hindered by worries of added cognitive and psychological impact on their own disease. As these patient volunteers navigate their own disease journey, they may struggle to retain complex protocols and guidelines. Unlike peer support programs for other diseases where recovery or cure is a common outcome, neuro-oncology survivors face a different trajectory that requires tailored support. Those with advanced illness may require increasing levels of support, while early-stage patients may confront the reality of disease progression, leading to anxiety about their own future.^15^ Given these challenges, it is crucial that neuro-oncology peer support programs prioritize ongoing monitoring and support of patient-volunteers’ emotional well-being. Additionally, many peer-to-peer support programs provide training for peer volunteers, but few provide continuous and formal support. Though this may not be necessary in types of cancer where patient volunteers show no evidence of disease or in community settings where patient volunteers are not under the care of the institution (eg American Brain Tumor Association CommYOUnity Connect program^16^) it is arguably warranted for peer volunteers who are patients within a neuro-oncology clinical setting.

Building on the growing need for continuous psychosocial support in neuro-oncology and intensified by the challenges posed by the SARS-CoV2 pandemic, our team at the University of California, San Francisco (UCSF) recognized an opportunity to create an innovative peer-to-peer support program. We launched the program in 2020, designed to provide ­structured peer support and continuous training for peer-volunteers while fostering a strong sense of community among survivors. This program served as a proof-of-concept for the effectiveness of peer volunteer programs in a clinical neuro-oncology environment. Herein, we report our initial observations from this program highlighting the conception, implementation and barriers, and propose a framework for building a successful peer-volunteer programs that can be integrated in other clinical neuro-oncology practices.

## Methods

The UCSF Neuro-Oncology peer-volunteer support program was developed in response to the emotional and mental well-being needs of patient volunteers, especially during the SARS-CoV-2 pandemic. To accommodate social distancing measures, we launched the program virtually and iteratively adapted its training materials and sessions.

The peer-to-peer program development process integrated existing peer-to-peer models, including the UCSF Cancer Center’s Peer Training Program^17^ and the UCSF Neuro-Oncology Caregiver Peer-to-Peer Program ^14^ as well as elements from the Cancer Coach Training program by the Cancer Journey Institute.^18^ These key elements included peer-volunteer training materials, peer matching protocols, and peer-volunteer support, which were adapted by the cancer coach trainer to better address the unique needs of brain tumor patients.

The program was initially conceived by a cancer coach trainer. For purposes of this program, the cancer coach trainer is defined as an experienced professional responsible for training peer supporters, health professionals, and community volunteers to effectively coach individuals affected by cancer (patients, survivors, and caregivers).^18^ Additionally, program development was supervised by the the Neuro-Oncology division chief and a nurse practitioner, who provided clinical guidance.

The initial structure of the program Included key members of the survivorship team:

- *Cancer Coach Trainer (CCT)*. Developed and oversaw the program, acted as a liaison between peer-volunteers (PVs) and peer-requestors (PRs), designed and adapted training materials, and facilitated the volunteer support group.
- *Neuropsychologist.* Provided psychological support to PVs on a referral basis, assisted the CCT in leading group sessions, and offered input to improve the program.
- *Oncology Social Worker*. Offered supplemental volunteer support and managed psychosocial risks, such as protocols for medical emergencies and suicidal ideation.
- *Program Assistant*. Recorded attendance, managed virtual session logistics, and created promotional communication materials.
- *Director of Neuro-Oncology Supportive Care Services*. Provided oversight and support for the personnel involved in developing the peer-to-peer program.
- *Clinical Providers*. Nurses, nurse practitioners, and neurooncologists who referred patients seeking support to this program and assisted in screening and identifying suitable PVs.
- *Peer-Volunteers (PVs)*. Screened and trained patients who provided support to peer requestors (PRs).

### Peer-Volunteer Selection

Potential PVs were identified through prior participation in a focus group or individual interview, via provider referral, or by self-referral in response to an announcement in an internal patient digital newsletter. Candidates were considered for training if they were: (1) a minimum of one-year post-diagnosis of a primary brain tumor; (2) able to perform ordinary tasks; (3) able to carry out their daily activities with minimal to no help; (4) reachable via an e-mail address; (5) able to fill out electronic applications; and (6) able to use virtual meeting software independently or semi-independently.

Candidates were asked to complete an interest form for demographics, contact information, and provider information. They were screened by the CCT involving at least one of the following: (1) referral from their neuro-oncologist or neuropsychologist; (2) in-person or phone-based interview with the CCT; or (3) direct observation of peer-to-peer interactions within patient groups and classes. Providers were contacted to identify any clinical or functional concerns that could interfere with the patient’s capacity to participate as a volunteer, which may lead to a temporary hold on their training invitation pending further review. Eligible candidates were then required to attend a two-hour virtual training session. This assessment was repeated annually to maintain compliance and relevance in addition to PVs choosing to place their participation on temporary or permanent hold.

Initial training sessions were offered yearly during the fall and consisted of a two-hour virtual group session. Training was designed around three guiding principles: (1) importance of confidentiality; (2) how to offer support and individual perspectives without administering medical advice or strong medical recommendations; and (3) self-care and ability to recognize situations requiring referral to the program’s staff.

Training was structured in two parts, each building on the other. The first part focused on the program’s mission and structure, an overview of expectations, confidentiality policies, and relevant community resources. The second part emphasized essential skills for peer support, including active listening, boundary-setting, and emergency protocols. The risk of emotional triggers that may be elicited in serving as PV was also emphasized. PV also had the opportunity to practice their acquired skill in small subgroups via scenarios and structured discussion guided by the CCT (Supplemental Table ST1).

After completing this initial training PV’s who remained interested and were deemed appropriate to participate completed a formal application of more detailed demographic information, self-reported diagnosis, treatment history, and topics that they were comfortable discussing with their peer matches. PVs signed a volunteer agreement outlining their role and the UCSF wide code of conduct while participating in the program, as well as a confidentiality agreement as per the UCSF’s institutional guidelines (Supplemental Materials SD4). PV’s information was maintained on a secure database managed by the CCT. At the end of each calendar year, volunteers were given the opportunity to continue their participation in the program and were required to sign a new confidentiality agreement form and update demographic and treatment information. PVs were allowed to pause or completely stop their participation at any time.

### Peer-to-Peer Matching Process

**Figure 1:**
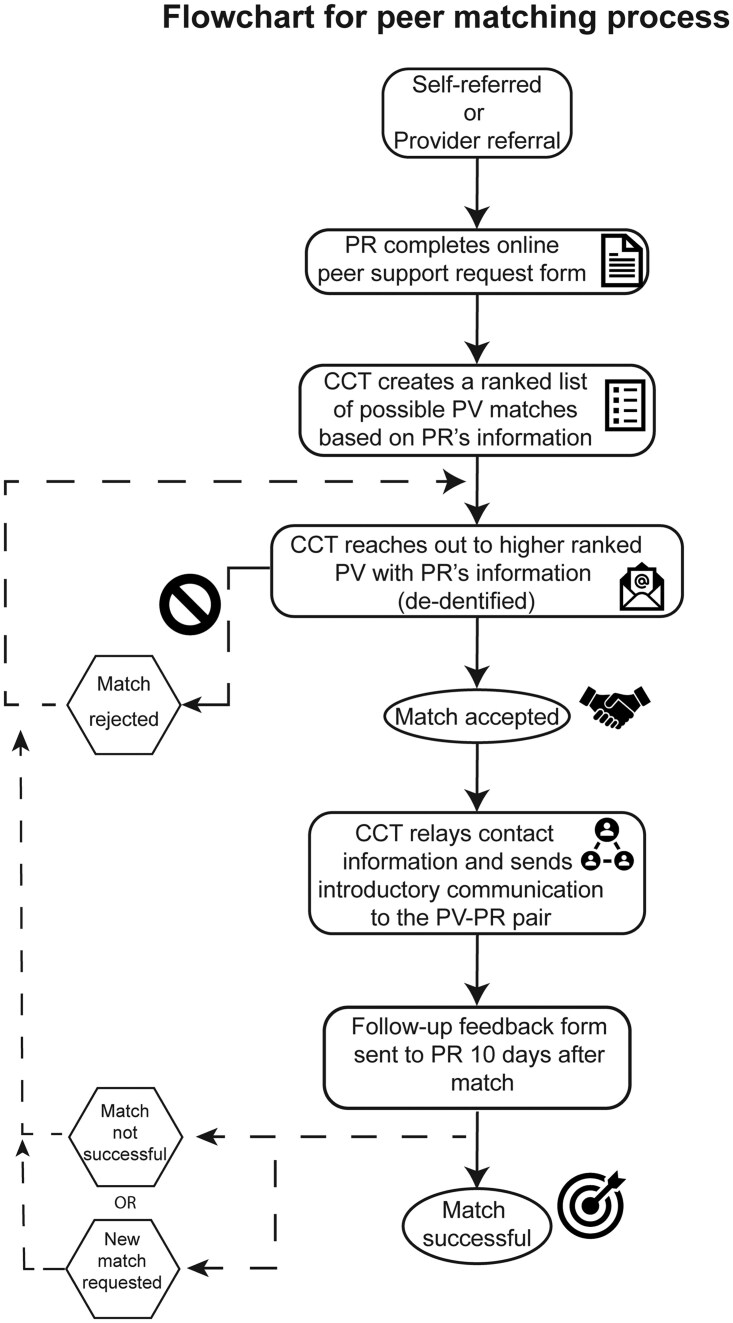
This flowchart outlines the steps for matching a patient requesting support (PR) with a suitable peer volunteer (PV). It depicts the initial request, screening, matching, and follow-up stages.

An electronic peer request (PR) form was created for patient inquiries for support and made available to any adult patient with a brain tumor diagnosis and advertised in the clinic waiting room and program’s website. Matching was performed within 48 hours of receiving the request. Matches were made by the CCT based on age, gender, relationship status, diagnosis, treatment history, psychosocial concerns, and intended discussion topic. After identifying potential matches for a request, suitable PVs were ranked by CCT and contacted sequentially via email and provided with anonymous PR information such as age, gender, and main topic of interest. They were given the opportunity to accept or decline a given PR match. If the PV declined a match, the next best match in the rank was contacted and so forth until a match was made (Figure 1). 

If the match request was accepted by the PV, contact information was shared in a separate email along with a reminder that their formal role is to engage in one conversation with the PR via phone or virtual encounter. Privacy measures were followed, and personal identifiers and health information in any electronic communication threads omitted in compliance with HIPPA guidelines.^19^ An introductory email was then sent to both the PR and PV to facilitate connection, and PVs were asked to notify the CCT when initial contact was made with their matched PR. Any additional encounters were at the discretion of the PR-PV pair. PVs were reminded to uphold the same code of conduct during all interactions, even those beyond their role requirement. PRs could request additional matches to further meet their needs. The CCT’s support was available throughout if any concerns or additional supportive needs arose. Informal collection of feedback from PRs and PVs allowed the CCT to identify areas of improvement in the matching process and overall program in an ongoing manner.

For purposes of these analyses, concerns expressed by requestors in open response to the question, “What would you like to discuss with a peer?” were categorized using an institutional artificial intelligence secured environment (UCSF Versa Application Programming Interface [API]). Within this secure environment, we made data requests to the GPT-4o model API. Parameters and prompt used for the classification can be found in Supplemental Materials SD1.

### Implementation of Peer-Volunteer Support Program (Thriver’s Program)

To keep volunteers engaged and provide additional support, a supplemental program was created consisting of an optional weekly one-hour PV meeting. Over time, PVs adopted the term “Thrivers” to reflect their own mindset towards their cancer journey and we refer to the weekly meetings as the Thrivers’ meetings. While participation was optional, all PVs were encouraged to attend any or all sessions at their own leisure. The objective of the meetings was to sustain a well-supported, skilled, and connected volunteer community capable of providing compassionate, reliable peer support to individuals living with brain cancer. As such, the meetings had several intentional goals including to:

- Support the matching process by maintaining contact with volunteers, allowing the CCT to deepen knowledge of each PV, assess their readiness for providing peer support, and stay updated on any changes in health or life circumstances that may affect their ability to fulfill their role.
- Provide emotional support for volunteers by regularly addressing the psychological and emotional burden of witnessing peers’ disease progression.
- Maintain volunteer engagement by offering opportunities to practice learned skills even during periods without active peer matches.
- Foster a supportive community that strengthens peer relationships and reduces volunteer isolation.

**Figure 2:**
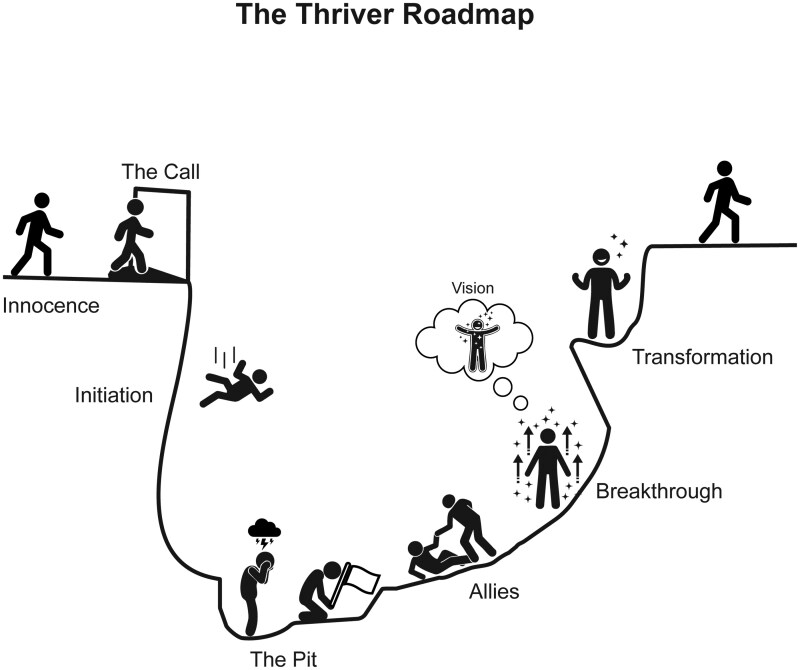
The Thriver Roadmap *(Adapted from the Cancer Journey Institute).* It illustrates the typical emotional and psychological journey of brain tumor patients. Beginning with **“The call”** at diagnosis, a moment often marked by shock and overwhelm due to the flood of clinical, social, and emotional information. During active treatment (**“Initiation”**), patients may struggle to keep up with all the rapid physical, cognitive, and lifestyle changes. Often when treatment ends, the expectation of life ever returning to ‘normal’ is challenged and many face a sense of despair, loss of identity and heightened isolation (**“The Pit”**). With time, grieving, gradual acceptance, and support from **“Allies”** (peers, therapists, and wellness resources), patients begin to reclaim their new identity, leading to a **“Breakthrough”**, marked by a deeper understanding of their strengths, resilience, values, and goals. This fosters a renewed **“Vision”** of life after diagnosis, culminating in **“Transformation”**, where patients recognize their personal growth. This cyclical process of adaptation and resilience can recur across the illness trajectory in response to evolving health or situational challenges (eg disease progression, job loss, etc.).

The program was led by the CCT with occasional guest speakers, which included a rehabilitation neuropsychologist, a social worker, and other relevant experts on topics of interest to the group. Thrivers’ meetings were conducted in three distinct formats:

1. Check-in meetings: PVs were led through semi-structured open discussions. The structured portion offered a self-assessment tool on a person’s social, physical, emotional, cognitive and spiritual (SPECS) well-being, ranking each item on a Likert scale from 1 to 10, where 10 represents the most positive attitude towards each area (see Supplemental Materials SF1). This tool offered participants a check-in framework, allowing opportunities to delve more deeply into any of the areas, particularly those assigned a lower score. Additionally, the sessions fostered an open environment where PVs could engage in unstructured conversations about challenges they faced in their peer-volunteer roles or openly share experiences from their own cancer journeys.
2. Mastermind sessions: Involved discussions about topics that were identified through polling PVs’ interests and occurred twice a year. Participants were asked “What would you like to discuss with a peer?” with open text responses. PVs were allowed to request multiple topics of discussion. Mastermind sessions took place monthly and the CCT facilitated the discussions using question prompts regarding the topic of interest. Mastermind sessions consider PVs as the experts in their own cancer journey, allowing them to share personal strategies that help them cope with difficult social, emotional, and practical situations. This not only served to help PVs individually address their current challenges but also provided them with collective wisdom to draw upon in support of their matched PRs during encounters.
3. Continuous Training sessions: Overarching goal of providing PVs with additional strategies and tools to help them in their volunteer role as well as in their own journey as brain tumor survivors (Thrivers). These tools included active listening, coping with their own emotional responses, and risk management protocols. Additionally, PVs were guided through a visual aid that helped them conceptualize the typical emotional cycle that a patient diagnosed with a brain tumor goes through during the different stages of disease (Figure 2).

### Program Evaluation

Feedback was obtained from PV’s informally and continuously during the sessions throughout 2020 and 2021. In 2022 and 2023, a formal survey was implemented to systematically gather anonymous feedback (Supplemental Materials SD2). This feedback was then shared by the CCT in annual meetings with key members of the survivorship program to discuss the program’s direction, challenges, proposed changes, and new implementations (if applicable).

## Results

### Peer-Volunteer Characteristics

Between August 2020 and December 2023, we identified 60 patients as potential PVs, with 48 (80%) training completion, and 46 (77%) enrolled in the program. The main reasons for not participating or enrolling in the program included conflicting priorities (*n* = 1) and not feeling emotionally ready to take on a supportive role (*n* = 1). One prospective PV had disease recurrence shortly after signing up for the training.

Mean age among included PVs was 48 years of age (SD 11.6); 52% were males, most PVs were married (65%), 56% had children, and most common self-reported histological tumor diagnosis was astrocytoma (33%) (Table 1).

**Table 1:** Characteristics of participants in the UCSF peer support program from 2020 to 2023, stratified by role as peer-volunteer or peer-requestor

Characteristic	Peer-volunteers (*n* = 46)	Patient-requestors (*n* = 116)
**Age, *mean* (SD)**	47.9 (11.6)	47.4 (13.3)
**Sex, *n (%)***		
Female	22 (48)	64 (55.2)
Male	24 (52)	49 (42.2)
**Marital status, *n (%)***		
Married/in a committed relationship	30 (65)	60 (52)
Single	10 (22)	43 (37)
Divorced or separated	3 (7)	8 (7)
Widowed	2 (4)	2 (1)
Prefer not to answer	1 (2)	3 (3)
**Have children, *n (%)***		
Yes	26 (56)	56 (48)
No	20 (44)	60 (52)
**Main clinical care at UCSF, *n(%)***		
Yes	44 (95)	94 (81)
No	2 (5)	22 (19)
**Diagnosis (self-reported), *n (%)***		
Astrocytoma	15 (32)	30 (26)
Glioblastoma	10 (22)	35 (30)
Oligodendroglioma	11 (24)	15 (13)
Meningioma	4 (9)	8 (7)
Other	6 (13)	10 (9)
Unspecified	0 (0)	18 (15)

**Table 2:** Breakdown of Concerns by Category in Response to the Question, “What would you like to discuss with a peer?”. Each concern entry can fall into multiple categories, reflecting the multifaceted nature of living with a brain tumor diagnosis. This table provides a clear overview of the distribution of concerns across the different categories, helping to identify key areas where support and resources may be needed

Category of topics to discuss with PV	Count of topics (*n* = 253)
1. Side effects and long-term effects of treatment	43 (16.9%)
2. Emotional and psychological impact	68 (26.9%)
3. Family and relationship dynamics	27 (10.7%)
4. Survivorship and quality of life	47 (18.6%)
5. Peer support and connection	37 (14.6%)
6. Practical and logistical concerns	22 (8.7%)
7. Other	9 (3.6%)

In the program’s first two years, approximately 75% of PVs engaged in 2 or more conversations (∼45 minute sessions) with their matched PRs, often remaining in contact beyond the required initial conversation. However, since 2023, this percentage has decreased to about 30%, reflecting a shift to mostly one-time conversations as the program progressed. In total, the team performed 132 peer matches for an average of 3 PRs for each PV.

### Patient-Requestor Characteristics

Between August 2020 and December 2023, 116 PRs reached out for peer-support. Ninety-four (81%) were patients from the UCSF Neuro-Oncology clinic while 22 (19%) received care elsewhere. The average age was 47 (SD = 13.3) years old, 55% were females, 52% were married, 48% had children, and the most common self-reported histological tumor diagnosis was Glioblastoma (30%) (Table 1).

### Peer-Requestor Interests and Concerns

The most common category among PRs to discuss with peer-volunteers were the “emotional and psychological impact of living with a brain tumor” (68.6%), “strategies for adjusting to survivorship and improved quality of life” (40.5%), and “side effect management and the long-term effects of treatment” (37.1%) (Table 2).

### Thriver’s Program

During the time analyzed, 175 weekly Thrivers’ meetings were conducted, with an average of 12 participants in attendance per session (Range = [3-22]). Seventy percent of all PVs attended 12 or more meetings per year.

Thirty PVs who participated in 2022 and 2023 Thrivers’ meetings provided feedback. Ninety-six percent (*n* = 29) reported to be extremely satisfied with the program, while only 4%(*n* = 1) reported to be somewhat satisfied with the program. Additionally, volunteers highlighted benefits such as mutual support, impactful contributions, and personal growth and empowerment. Of note, volunteers who reported feeling inadequately resourced to engage in peer-support conversations attended <12 Thrivers’ meeting sessions per year.

Open ended survey questions regarding the utility of the program highlighted “the value of hearing from someone with a shared experience” and enhanced emotional well-being as a result of the Thrivers’ program (Supplemental Table ST2). Based on further review of the PV’s feedback, additional topics were integrated into the curriculum, including a refresher on responding to medical emergencies (eg seizures), as well as the creation of a structured format for volunteers to share their own best practices for supporting peers and for living well (mastermind sessions).

Recognizing the strong social bonds that had formed in these weekly meetings, we accommodated the desire of PVs to continue to participate in Thrivers’ activities even if their status as a peer volunteer was on hold (ie removed from the matching pool). Though not a common occurrence, this allowed a patient to remain a ‘Thriver’ regardless of their active participation as a PV.

PVs often referred back to the Thriver Roadmap tool ­(Figure 2), verbalizing when they or their PR match were in the “Pit” of their journey. Several members identified this visual tool as an accurate representation of their overall process of shifting their identity from ‘Survivor’ to ‘Thriver’.

While the presence of clinical staff during some meetings was appreciated due to their professional perspectives and their insight into clinical operations, a few Thrivers expressed feeling more comfortable in an environment without healthcare team members present, allowing them greater freedom to communicate their experiences and challenges. Without the presence of healthcare professionals, some participants felt more empowered as “experts in their own experience”. They could confidently share their unique perspectives from their personal cancer journeys, creating an egalitarian atmosphere where each voice was valued. This dynamic enriched peer-to-peer learning and support, and underscored the importance of centering the patient perspective in these meetings.

### Lessons Learned

The development and implementation of our peer-to-peer support program revealed a range of challenges and corresponding adaptations that have shaped its evolution. Structured support and continuous adaptation were crucial for the well-being of volunteers, who reported high levels of satisfaction and a strong sense of community and personal fulfillment. While the core aim remains to support patients with brain tumors, our experience underscored the importance of also addressing the needs of PVs. Through targeted training, ongoing support, and the creation of a dedicated community space (Thrivers), the program has promoted both resilience and fulfillment among PVs. Key adaptations such as virtual delivery, refining request processes for better data capture, and establishing clear boundaries through training and conduct policies, have contributed to the program’s long-term sustainability, safety, and participant satisfaction. Feedback from participants further informed adjustments such as the introduction of a structured curriculum, accessible emergency preparedness protocols, and identity-affirming practices, which have enhanced the program’s effectiveness and relevance. These insights reflect a model of continuous improvement that prioritize patient-centered care while recognizing the complex emotional landscape of survivorship. A summary of program elements, associated challenges, program adaptations, and lessons learned is provided in Supplemental Table ST3.

## Discussion

Patients living with brain tumors have unique and complex needs that encompass both medical treatment and the emotional and psychosocial impacts of their disease. Timely and effective support throughout their disease journey is crucial. In alignment with current practices of supportive care, we have shown that peer-volunteer programs require continuous iterative processes of improvement to enhance the services offered to participants.^15^^,^^20^^,^^21^ A key insight that emerged from our efforts was the need for additional support for the peer-volunteers themselves, given the dual burden of their own disease and the emotional toll of supporting others.

By engaging in roles to support their patient-peers, PVs develop a mindset centered on resilience, purpose, and empowerment. Interacting with like-minded individuals fosters a sense of community and helps PVs reshape their identity to embrace both their challenges and coping strategies. This shift has led the group to adopt the term “Thrivers” reflecting their view of themselves as active participants in their own well-being and enabling them to share that knowledge and experience to others.^11^ Volunteering while living with a brain tumor creates a unique identity among survivors, distinguishing them from the broader patient group. Their focus extends beyond their own experience to learning and offering coping strategies to help others.

Structured support can help PVs refine and develop their new identity. Our team provides this support through “Thrivers’ meetings” guided by the CCT. Unlike general support groups, these discussions focus on developing skills and best practices for personal growth and community service. This aspirational label is not dependent upon externally measurable standards and is applied to all patients who achieve peer volunteer status. It affords PVs who experience disease progression or health setbacks to remain a “Thriver” in the eyes of other members as well as in the eyes of the CCT.

Sustaining such peer-to-peer support programs, however, requires careful attention to both individual and structural factors. On an individual level, the long-term involvement of PVs may not always be feasible due to changes in health status or personal circumstances. Structurally, ensuring program continuity depends on stable financial support and ongoing community engagement. To address these challenges while preserving the integrity of the PR-PV connection, our program implemented practical measures such as the “single encounter expectation” and the “three call rule.” These guidelines were designed to support healthy boundaries, respect patient autonomy, and mitigate the risk of emotional burnout among volunteers, thereby promoting sustainability for both the individuals involved and the program as a whole. While these measures have proven effective within our program and patient population, their continued success requires ongoing input from both PRs and PVs, along with a comprehensive assessment of the group’s evolving needs and available resources.

To ensure community sustainability, it is essential to recognize and cultivate the intrinsic motivation of peer volunteers. We have observed that when volunteers feel valued and supported, they are often eager to take on greater roles within the program. This presents an opportunity to deepen their engagement through targeted training that enhances their communication skills, builds confidence, and reinforces their identity as vital contributors to the program. By aligning training efforts with volunteers’ personal motivations and lived experiences, peer-to-peer programs can encourage long-term volunteer retention and a stronger sense of ownership. To achieve this, training should be thoughtfully tailored not only to reflect the interests and goals of PVs, but also to prepare them for the interpersonal challenges that may arise when engaging with PRs while maintaining professional standards. These adaptations should be informed by the program team’s ongoing monitoring of PR-PV interactions, balancing the need for support and quality assurance with the importance of preserving the privacy and autonomy of these relationships. Additionally, we have observed that involving key PVs in advisory roles to help shape the program and contribute to educational materials for the survivorship program is mutually beneficial. PVs are able to see an immediate impact of their contributions, which further cultivates the feeling that their voice matters.

Equally important to sustaining peer-to-peer programs is the presence of strong institutional support. Engaging stakeholders early and consistently is essential, not only to advocate for the program’s value, but also to ensure its long-term viability. Demonstrating impact through a combination of objective outcomes and patient or volunteer narratives can be a powerful strategy to garner continued investment. This collaborative, evidence-informed approach reinforces the foundation of peer support initiatives and improves the likelihood of securing the financial and structural resources necessary for sustained impact.

Looking ahead, a key priority for the future of this program is the systematic collection of data to rigorously evaluate its benefit and impact, both from the perspective of the PR and the PV. While early efforts must prioritize demonstrating financial and institutional viability, the long-term success of peer-to-peer support depends on our ability to link participation with meaningful outcomes. This includes not only improvements in emotional and psychosocial well-being, such as reductions in distress and increases in hope, empowerment, and sense of purpose, but also potential effects on objective, measurable clinical outcomes such as treatment adherence, symptom improvement, and ultimately enhanced quality of life. To that end, future research should incorporate structured data collection from PVs, PRs, and healthcare providers, enabling a formal and rigorous assessment of changes in quality of life, symptom burden, care navigation success, and health-related decision-making. Capturing this data validates the programs’ effectiveness and provides a reproducible framework that can be adapted, integrated into clinical care, and scaled across diverse health systems.

As noted previously, the original motivation for this program emerged during the COVID-19 lockdown, which left many patients living with brain tumors and caregivers feeling isolated. In that context, the virtual format provided an essential lifeline that allowed participants a way to connect with a community of like-minded individuals, share lived experiences, and foster mutual support. As we move beyond the constraints of the pandemic, there is a valuable opportunity to build on these virtual foundations using in-person events that further enhance the impact. Annual in-person retreats focused on volunteers, for example, present promising avenues not only to reinforce the sense of community but to create stronger links among this community. These gatherings can serve multiple purposes: providing additional training and face-to-face practice opportunities, reinforcing communication and empathy skills, and, perhaps most importantly, cultivating a sense of belonging and shared identity among PVs. Such events can further reduce feelings of isolation, reinforce empowerment, and help embody the “Thriver” identity at the heart of the program. By celebrating community, resilience, and purpose, these in-person events can play a critical role in sustaining motivation and cohesion within the peer network.

As efforts continue to strengthen the community aspect of the program through meaningful encounters, it is important to consider how the program can scale efficiently without losing its core values. While peer-to-peer support programs offer immense value, they also require significant coordination across multiple fronts, including recruitment training, PR-PV matching, and ongoing engagement. To expand the program’s reach and serve a broader population, one proposed strategy involves engaging experienced PVs to eventually take on facilitator roles. In parallel, it is essential to explore tools that can streamline specific aspects of the workflow. However, such tools should be implemented only after careful evaluation of at least two key criteria: (1) the step is essential to the program’s functioning, and (2) it can be automated with minimal -but not entirely absent- oversight from staff and stakeholders. When applied thoughtfully, these tools can enhance efficiency and maximize the use of limited resources without compromising the patient-centered nature of the program.

For example, our group has begun exploring the use of an AI-informed match generation process, which allows for more dynamic, comprehensive, and responsive pairing between PRs and PVs. Unlike traditional approaches that rely on the staff to recall each volunteer’s experiences and strengths, a task that becomes increasingly challenging as the community grows, this tool allows real-time adjustments to relevant matching variables, thereby improving the precision and quality of each connection. By leveraging modern technologies such as large language models, we aim to ensure that the program remains scalable and sustainable, preserving the depth of the peer-to-peer relationship while expanding its reach to benefit more patients.

Together, these training, community-building, and technological integrations represent important steps toward a more resilient and scalable peer-to-peer support model. As the program continues to evolve, maintaining a balance between human connection and operational efficiency will be key.

## Conclusion

With the foundations of our model for a peer-to-peer support program, we have observed that supporting volunteers through structured programs yields benefits not only for the individuals involved but also for the broader community they help sustain. These efforts set the stage for a more robust survivorship model, which recognizes the reciprocal nature of peer support and the importance of empowering volunteers in our brain tumor survivor community.

Within the UCSF Neuro-Oncology division, our Peer Support Program has demonstrated strong feasibility, engagement, and preliminary benefits for volunteers. While peer-to-peer programs are set up to provide support to patients who need it, we emphasize that volunteers have their own needs, and we have observed that providing volunteers with adequate tools and skills to face different situations while supporting their peers is fundamental to the program’s success in brain tumor populations. The peer-to-peer service and Thriver community lie at the heart of our survivorship program, embodying our mission to foster resilience, connection, and hope among those navigating life after a brain tumor diagnosis. By addressing emotional isolation and providing an avenue for meaningful community engagement, the peer volunteer program offers a potentially replicable model to other institutions seeking to enhance survivorship care in brain tumor populations. Further systematic data gathering and research aimed at measuring the impact of the program on clinical and quality of life outcomes of PVs and PRs, especially in the longitudinal setting, will help solidify best practices and ensure the continued effectiveness and sustainability of this innovative approach.

## Supplementary Material

npaf119_Supplementary_Data

## Data Availability

The data that support the findings of this project are available from the corresponding author upon reasonable request. Restrictions may apply to the availability of some data due to privacy or ethical considerations.
