# Supplementary material for: Creating a neuro-oncology framework for an empowered and engaged peer volunteer patient community
Source: Neurooncol Pract. 2025 Nov 18;13(2):363–72. doi: 10.1093/nop/npaf119 (PMC13153703; doi:10.1093/nop/npaf119)
Supplement: npaf119_Supplementary_Data [file npaf119_supplementary_data.zip › SD1-LLM_Parameters_Categorize_Topics.docx]

**Supplemental Document SD1. Large Language Model Parameters and Prompt Used to Categorize Topics of Interest Among PRs**

parameters = {model = gpt-4o, temperature = 0.7, n = 1,

prompt = f"""

You are a helpful expert in classifying unstructured data. A subject is expressing the topics they are interested in discussing: "{text}".

Classify their response into one or more of the following categories.

If multiple topics are mentioned, separate them with a comma.

**Categories:**

1. **Side Effects and Long-Term Effects of Treatment** → Anything related to **physical health impacts, treatment side effects, or long-term health challenges**.

2. **Emotional and Psychological Impact** → Feelings, anxiety, depression, coping mechanisms, therapy, trauma.

3. **Family and Relationship Dynamics** → Topics related to **family struggles, relationship changes, support from loved ones**.

4. **Survivorship and Quality of Life** → Life after treatment, adjusting to normal life, post-treatment struggles.

5. **Peer Support and Connection** → Talking with others who have similar experiences, patient groups, mentorship.

6. **Practical and Logistical Concerns** → Insurance, work, financial struggles, travel for treatment, navigating the healthcare system.

7. **Other** → Anything that does not fit above.

# Few shot approach

**Examples**

- "Supporting my family support team" → **Family and Relationship Dynamics**

- "How do I deal with the emotional trauma of this diagnosis?" → **Emotional and Psychological Impact**

- "I just want to meet people who understand my journey." → **Peer Support and Connection**

- "Insurance paperwork is overwhelming." → **Practical and Logistical Concerns**

- "The side effects of chemo are terrible." → **Side Effects and Long-Term Effects of Treatment**

- "Getting back to normal life after treatment is tough." → **Survivorship and Quality of Life**

- "I want to talk about something else entirely." → **Other**

**Instructions**

- Return **only** the category names, separated by commas if multiple apply.

- If the response does not fit any of the six main categories, return the categories that you think would be.

"""
