# Supplementary material for: Creating a neuro-oncology framework for an empowered and engaged peer volunteer patient community
Source: Neurooncol Pract. 2025 Nov 18;13(2):363–72. doi: 10.1093/nop/npaf119 (PMC13153703; doi:10.1093/nop/npaf119)
Supplement: npaf119_Supplementary_Data [file npaf119_supplementary_data.zip › ST1-Peer_Volunteer_Training_Agenda_Sample.docx]

**TABLE ST1. Sample of Peer Support Training Agenda**

| **Agenda Item** | **Duration** |
| --- | --- |
| Welcome & introductions | 15 minutes |
| Program overview and purpose | 5 minutes |
| Role of the volunteer | 10 minutes |
| Logistics – how we match | 5 minutes |
| Helpful local, national and international resources | 10 minutes |
| Structure of a peer conversation and sample script | 5 minutes |
| Break | 5 minutes |
| Listening skills & empathy | 15 minutes |
| Sharing your experience | 5 minutes |
| Experiential exercise: role plays & discussion | 20 minutes |
| Self-care and when to contact the staff | 5 minutes |
| Review: Volunteer do’s and don’ts | 5 minutes |
| Questions and concerns | 10 minutes |
| Next steps and Thrivers Group | 5 minutes |
| **Total** | **120 minutes** |
