# Supplementary material for: Creating a neuro-oncology framework for an empowered and engaged peer volunteer patient community
Source: Neurooncol Pract. 2025 Nov 18;13(2):363–72. doi: 10.1093/nop/npaf119 (PMC13153703; doi:10.1093/nop/npaf119)
Supplement: npaf119_Supplementary_Data [file npaf119_supplementary_data.zip › ST2- Thrivers Feedback.docx]

**Supplemental Table ST2. Thriver’s Program Feedback**

| **How satisfied are you with the training you have been given to prepare you for the role of peer volunteer?** | **What do you appreciate most about the Peer Volunteer & Thriver program?** |
| --- | --- |
| Extremely satisfied | The support and openness of everyone. Also that I can still support, have an impact, and not take up so much time. |
| Extremely satisfied | Naomi’s content and all the thrivers participation |
| Somewhat satisfied | The sense of being a part of a strong, caring community that accepts me, loves me, and where what I have to share is meaningful, at least on some occasions, to others. |
| Extremely satisfied | The camaraderie, and like experience in a unique, niche situation. |
| Extremely satisfied | Give us a place to talk over our feelings with others in a similar situation. |
| Extremely satisfied | Listening to other patient's experieces. |
| Extremely satisfied | Connections with people who have similar experiences |
| Extremely satisfied | The leadership! |
| Extremely satisfied | Attitude of acceptance for being vulnerable, emotional, ill, etc. |
| Somewhat satisfied | The support from the group. |
| Extremely satisfied | I really enjoy my current match with a peer support thriver. We have connected and talked and have helped each other greatly. I think this relationship is invaluable to both of us! |
| Extremely satisfied | naomi's skill and personality that gives positive feedback where appropriate and constructive suggestuis where helpful |
| Extremely satisfied | Creating safe and encouraging relationships with my peers, mentors and mentees. |
| Extremely satisfied | Cancer and cancer connecting. Almost all of us speak with counselors, but it's so nice to be able to speak with someone who suffers as well; there's a unity that's difficult to describe. |
| Extremely satisfied | I most appreciate Naomi and Mary’s thoughtful, loving and skilled facilitation. It has allowed us to build a really safe and special place. This is especially relevant as we have lost 3 thrivers this year alone. |
| Extremely satisfied | The relationships built are most important |
| Extremely satisfied | The guidenance get from Naomi, Mary and Lacy is incredible and I really appreciate having access to their expetise and compassion. I also learn from the other Thrivers and lean on them when I need support. |
| Extremely satisfied | Very, very friendly and welcoming and smart |
| Extremely satisfied | The sense of camaraderie, connection, and community. The expert facilitation. The unique opportunity to practice powerful life-changing tools in a safe container (like breakouts) with people who understand the experience of living with brain cancer. |
| Extremely satisfied | Opportunity to help and support others. Helps me process and reflect on my own experience and circumstances. |
| Extremely satisfied | The support as a whole. The ability to express myself and to hear others do the same are both very important. Learning from others is key. I'm also passionate about trying to help others so this is great for me and I'm learning a ton on how to better do so. |
| Extremely satisfied |  |
| Extremely satisfied | Being in community with others who truly understand all the ways that brain cancer changes and shapes our perspectives is invaluable. Having an opportunity to share with each other how we've weathered the multitude of challenges we face, to hear what helped others, and to transform the 'crappy hand' we've been dealt into something good - to support and encourage others in their seasons of difficulty - is life-changing. |
