# Supplementary material for: Creating a neuro-oncology framework for an empowered and engaged peer volunteer patient community
Source: Neurooncol Pract. 2025 Nov 18;13(2):363–72. doi: 10.1093/nop/npaf119 (PMC13153703; doi:10.1093/nop/npaf119)
Supplement: npaf119_Supplementary_Data [file npaf119_supplementary_data.zip › ST3-Challenges_and_Lessons_Learned.docx]

**Supplemental table ST3. Summary of program elements, associated challenges, program adaptations and lessons learned**

| **Program Element** | **Challenge/Observation** | **Resolution (Implementation and Adaptation)** | **Lesson Learned** | **Feedback and Outcomes** |
| --- | --- | --- | --- | --- |
| **Patient Support** | Fast access to professional emotional support for patients is limited, often with long wait lists. | Enlist stable patients to provide support to fellow patients. | With adequate training and support for themselves, PVs can provide helpful emotional support to fellow patients in mild to moderate distress. | Leveraging the time and efforts of survivors motivated to “give back” can significantly enhance the emotional support available to patients. |
| **Patient Requests and Matching Process** | Difficult to scale the number of match requests at a given time. | Modify intake forms to offer structured selections instead of open-text fields. | Structured selections improve efficiency and consistency in data collection. | Improved matching process efficiency and consistency. Supported program improvements and data organization for future analysis. |
| **Thrivers Meetings** | Addressing emotional burden and burnout among volunteers. | Weekly meetings led by CCT, occasionally co-facilitated by a social worker and neuropsychologist. Structured curriculum alternating training, discussions, and check-ins. | Regular, structured support helps manage emotional burden and fosters community. | Participants expressed high satisfaction with the sense of community and contribution. Thrivers presentations improved communication skills. |
| **Training Curriculum and Meeting Structure** | Diverse needs and motivations among PVs. | Year-long alternating curriculum; pre-distribute materials and discussion prompts. | Flexible, varied content maintains engagement and skill development. | Improved meeting participation and content retention. |
| **Identity and Role Evolution** | PVs felt misidentified as general survivors | Adopt the term 'Thrivers'; encourage leadership and personal expression. | Language and identity shape engagement and empowerment. | Strengthened PV identity; inspired independent support initiatives. |
| **Thriver Member Contact and HIPAA** | Fostering connection among PVs while respecting privacy. | Create and share an ‘opt-in’ PV member contact list. Suggest direct PV-PV contact for support and added community. | PVs who regularly attend meetings form deep connections. Direct contact can offer fast peer support among PVs. | PVs form individual relationships which offer added support for themselves, for their PRs, and which foster additional creative efforts and patient advocacy. |
| **Managing Unresponsive Peer Matches** | Shifting health and priorities of PRs may make PR-PV contact difficult. | Implement a ‘three-call-rule’ for volunteers to disengage after three unanswered contacts. | Setting clear guidelines helps protect volunteers from overburden and PRs from feeling harassed if their desire for contact changes. | PVs appreciated this guideline to relieve their sense of responsibility. |
| **Single Encounter Expectation** | Volunteers might experience cumulative burdens and emotional distress from repeated encounters. | Emphasize the one-call expectation with a written reminder in the email to both parties | Setting clear expectations helps maintain healthy boundaries and protect volunteers. | Volunteers better manage emotional distress and maintain healthy boundaries. |
| **Emergency Preparedness** | Equip volunteers to appropriately respond to medical or psychological emergencies occurring during sessions. | Provide visual diagrams, institutional emergency contact information, and sample scripts for communication. Use Thrivers meetings for sharing experiences and requesting resources. | Clear protocols and ongoing training empower volunteers to respond appropriately. | Volunteers feel ready to handle emergencies while maintaining boundaries. |
| **Code of Conduct** | Maintaining professionalism and appropriate boundaries in evolving PV-PR friendships. | Annual review and electronic signing of Code of Conduct and scenario-based training. | Regular reaffirmation and training on professionalism are essential. | Ensures volunteers provide emotional support while safeguarding peer support integrity. |
| **Sustainability and Scalability** | High time and staffing demand for managing weekly volunteer program. | Program requires a dedicated survivorship program manager. Consider supporting and training senior PVs to take on facilitation role. | Resource investment is critical for sustainability. | Framework shows promise for expansion; requires institutional and community support. |
| **Independent Efforts and Initiatives** | Volunteers want to extend support beyond the program. | Support leadership and innovation among volunteers. | A recognized and valued peer support volunteer role empowers patients to create their own communities of peer support. | Inspired independent peer support programs and NGOs. Proposed peer-led Young Adults with Brain Tumors support group. |
