# Supplementary material for: Creating a neuro-oncology framework for an empowered and engaged peer volunteer patient community
Source: Neurooncol Pract. 2025 Nov 18;13(2):363–72. doi: 10.1093/nop/npaf119 (PMC13153703; doi:10.1093/nop/npaf119)
Supplement: npaf119_Supplementary_Data [file npaf119_supplementary_data.zip › SD2-Feedback_Peer_Volunteer_Survey_sample.pdf]

Feedback survey form regarding Patient-Volunteer (PV) experience

Q8. Thank you for taking the time to answer these questions about the **Peer Volunteer Program and Thriver Community**. Your responses are anonymous and will be used collectively for quality improvement and reporting purposes.

Q14. How satisfied are you with the **training** you have been given to prepare you for the role of peer volunteer?

Extremely satisfied

Somewhat satisfied

Neither satisfied nor dissatisfied

Somewhat dissatisfied

Extremely dissatisfied

Q2. How adequately prepared do you feel for your role as a peer volunteer?

Extremely well prepared

Somewhat well prepared

Somewhat unprepared

Extremely unprepared

Q1. How many peers have you been matched with since your training?

I have not been matched yet

1 - 2 matches

3 -4 matches

5 or more matches

Q7. If you have had a peer match, please state the nature of your peer relationship (choose more than one if it has been different for separate peer matches):

SAMPLE

We had one in depth conversation

We had one conversations and I would check in periodically with no reply

We had several conversations and check-ins

We have remained in contact and have become close

Other

Q3. What areas would you like additional training in, if any?

Q4. How many Thursday Thriver meetings have you attended?

2 or fewer meetings

3 - 10 meetings

11 - 30 meetings

over 30 meetings

Other:

Q12. If you have not attended many meetings please indicate your primary reason(s) (choose all that apply):

I plan to attend more but have just recently joined as Peer Volunteer

The time conflicts with other important commitments

It is too depressing or frightening to hear about other people's problems

I have been too ill or have not had the energy to attend

I do not wish to spend any more time than necessary focusing on my brain cancer

I do not trust this format for keeping my information private

I see no benefit to attending these meetings

Other (please indicate):

Q5. What do you **appreciate most** about the Peer Volunteer & Thriver program?

Q6. What do you think **could be improved** about the Peer Volunteer & Thriver program?

Q10. What is your **overall level of satisfaction** with the UCSF Neuro-Oncology Peer

SAMPLE

Volunteer & Thriver Community?

Extremely satisfied

Somewhat satisfied

Neither satisfied nor dissatisfied

Somewhat dissatisfied

Extremely dissatisfied

Q11. Please use this space for any additional comments:

SAMPLE
