# Supplementary material for: Creating a neuro-oncology framework for an empowered and engaged peer volunteer patient community
Source: Neurooncol Pract. 2025 Nov 18;13(2):363–72. doi: 10.1093/nop/npaf119 (PMC13153703; doi:10.1093/nop/npaf119)
Supplement: npaf119_Supplementary_Data [file npaf119_supplementary_data.zip › SD4-Volunteer Agreement.pdf]

Volunteer Agreement

Please read the following Agreement for Peer Support Volunteers:

As a peer support volunteer of the UCSF Peer Support Program through the UCSF Neuro-Oncology Survivorship Program, I agree to:

- Uphold the UCSF Ethical Standards and Code of Conduct as outlined here.
- Complete all the training required of this program.
- Maintain the values, skills and sensitivity emphasized in the training curriculum.
- Regard the patient with dignity and worth at all times.
- Not dispense medical advice.
- Maintain the confidentiality of all the patients I speak with through the Peer Support Program and Thrivers Group.
- Report back to the Peer Support Program whether I have connected with a patient I have been matched with.
- Report back to the Neuro-Oncology Survivorship Program immediately on any observations relating to the patient's well-being that are of concern, especially if plans to harm self or others are indicated.
- Complete the UCSF Volunteer HIPAA training component, sign the UCSF Confidentiality of Patient, Employee and University Business Information and Computer Use Agreement and provide original signed copy to my Peer Support Administrator prior to receiving any patient information.
- Allow my personal information to be entered into a secure database for the purposes of matching me with a patient. I understand that my first name will be shared with my match prior to my contact with them. Other information about my medical or personal situation may be implied based on the requestor's needs for an appropriate match.
- Complete my time commitment of one year unless there is a change in my status as a patient and/or I do not feel that my services would be beneficial as a peer support volunteer to a patient.
- Notify the Survivorship Program if I choose to no longer participate in the peer support program. Once notice of withdrawal is received by program staff, my personal and/or medical information will no longer be provided to peer matches.

- Failure to comply with these guidelines may result in the revocation of my volunteer status.

I have read this consent and agree ________________________________

Date __________________________

SAMPLE
